# Novel low-sodium salt formulations combined with Chinese modified DASH diet for reducing blood pressure in patients with hypertension and type 2 diabetes: a clinical trial

**DOI:** 10.3389/fnut.2023.1219381

**Published:** 2023-09-07

**Authors:** Ziyan Zhang, Xiaomeng Zhou, Ying Mei, Xiaoqing Bu, Jie Tang, Tao Gong, Guowei Liu, Shuwen Cai, Yanni Ren, Lihong Mu

**Affiliations:** ^1^Department of Epidemiology, School of Public Health, Research Center for Medicine and Social Development, Chongqing Medical University, Chongqing, China; ^2^Center for Disease Control and Prevention, Fengjie County, Chongqing, China; ^3^Health Management Center of the Second Affiliated Hospital of Chongqing Medical University, Chongqing, China; ^4^Department of Nutrition and Food Hygiene, School of Public Health, Chongqing Medical University, Chongqing, China; ^5^Center for Disease Control and Prevention, Nan’an District, Chongqing, China

**Keywords:** blood pressure, DASH diet, hypertension, low-sodium salt, type 2 diabetes

## Abstract

**Background:**

In this study, we aimed to explore the antihypertensive effect of 23 and 52% concentrations of low-sodium salt combined with the Chinese Modified Dietary Approaches to Stop Hypertension (CM-DASH) diet in patients with hypertension and type 2 diabetes.

**Methods:**

We conducted a randomized controlled single-blind trial with a semi-open design. One hundred and thirty-two participants were randomly assigned into Group A (control group), Group B (52% low-sodium salt group), Group C (23% low-sodium salt group), and Group D (meal pack group) for 8 weeks of dietary intervention. All participants were followed weekly to collect data on blood pressure, salt use, and adverse events. Blood and 24-h urine samples were analyzed at baseline, 4 weeks, and the end of the intervention.

**Results:**

At the end of the intervention, the mean blood pressure decreased significantly by 10.81/5.03 mmHg, 14.32/6.32 mmHg, 14.20/6.59 mmHg, and 19.06/7.82 mmHg in Groups A–D, respectively, compared with baseline (*p* < 0.001). Comparison between groups showed that the systolic blood pressure was lower in Groups C and D than in Groups A (−6.54 mmHg, −8.70 mmHg, *p* < 0.05) and B (−6.60 mmHg, −8.76 mmHg, p < 0.05), and the diastolic blood pressure was lower in Group D than in Group A (−5.17 mmHg, *p* = 0.006). The 24-h urinary Na^+^ and Na^+^/K^+^ values were significantly decreased in participants using low-sodium salt (*p* < 0.001). No serious adverse events occurred during the trial.

**Conclusion:**

Our preliminary results suggest that 23 and 52% concentrations of low-sodium salt combined with the CM-DASH diet can effectively reduce sodium intake and increase potassium intake in patients with hypertension and type 2 diabetes mellitus, thus achieving “salt reduction” and attaining standard, smooth, comprehensive management of patients with hypertension and type 2 diabetes.

**Clinical trial registration:**

http://www.chictr.org.cn/, ChiCTR2000029017.

## 1. Introduction

Hypertension and diabetes are common chronic non-communicable diseases worldwide (1), and are often complicated by each other because they have the same causes and serve as mutually influential factors. A Chinese nationwide cross-sectional survey showed that 27.9% of patients had type 2 diabetes, 59.8% of which were complicated with hypertension (2). Patients with comorbid hypertension and diabetes have a significantly increased risk of cardiovascular and cerebrovascular events (3) and greater renal impairment than those with hypertension or diabetes alone (4). Several studies have shown that imbalance in daily dietary pattern and the content of sodium and potassium in food can directly affect blood pressure, which may be related to the effect of sodium on hemodynamics (5, 6). Hypertension and diabetes are both related to lifestyle; thus, lifestyle interventions are integral to disease control, and dietary intervention is an important link in life intervention. A balanced dietary pattern with lower sodium and higher potassium has interested researchers. In particular, an eating plan that originated in the United States, the Dietary Approaches to Stop Hypertension (DASH) diet, has shown promising results in lowering blood pressure in both normo-and hypertensive patients, which is attributed to the recommended foods that are rich in potassium, low in sodium, and high in dietary fiber (7, 8). However, promotion of the DASH diet in China has been challenging, as the diet plan entails restriction of salt intake, and most Chinese individuals prefer high-salt foods (i.e., average salt intake of 12 g/day) (9).

As a result, research on the use of low-sodium salts has emerged. Low-sodium salt is ordinary table salt (90–99% sodium chloride) mixed with varying proportions of potassium chloride (KCl), which also has a salty flavor, thereby reducing sodium chloride (NaCl) intake. A 5-year trial in China confirmed the health benefits and safety of low-sodium salt; however, the trial did not assess blood potassium markers, and it was unclear whether hyperkalemia could be a consequence (10). A review showed that low-sodium salts slightly increased blood potassium by 0.12 mmol/L, and lowered systolic and diastolic blood pressure by an average of 4.76 mmHg and 2.43 mmHg, respectively (11). In addition, the use of low-sodium salts varies widely depending on the region and tastes, e.g., developed countries, such as the United States, may produce foods with 100% potassium chloride, whereas India produces foods with only 20%, and in China the content of potassium chloride is 25 to 30% (12, 13). As such, very few trials have been conducted in China using 23% versus 52% low sodium salt (NaCl content) in combination with the modified DASH diet.

Therefore, in this trial, we used two low-sodium salt concentrations (52 and 23% NaCl) and a meal pack to explore the effects of their combined intervention with the Chinese Modified (CM)-DASH diet on reducing the blood pressure, reducing sodium intake, and increasing potassium intake in patients with hypertension and type 2 diabetes.

## 2. Materials and methods

### 2.1. Sample size

Based on the pretest data estimation (14, 15), 30–40 cases were included in each group, totaling approximately 120–150 cases.

### 2.2. Participants

**Figure 1:**
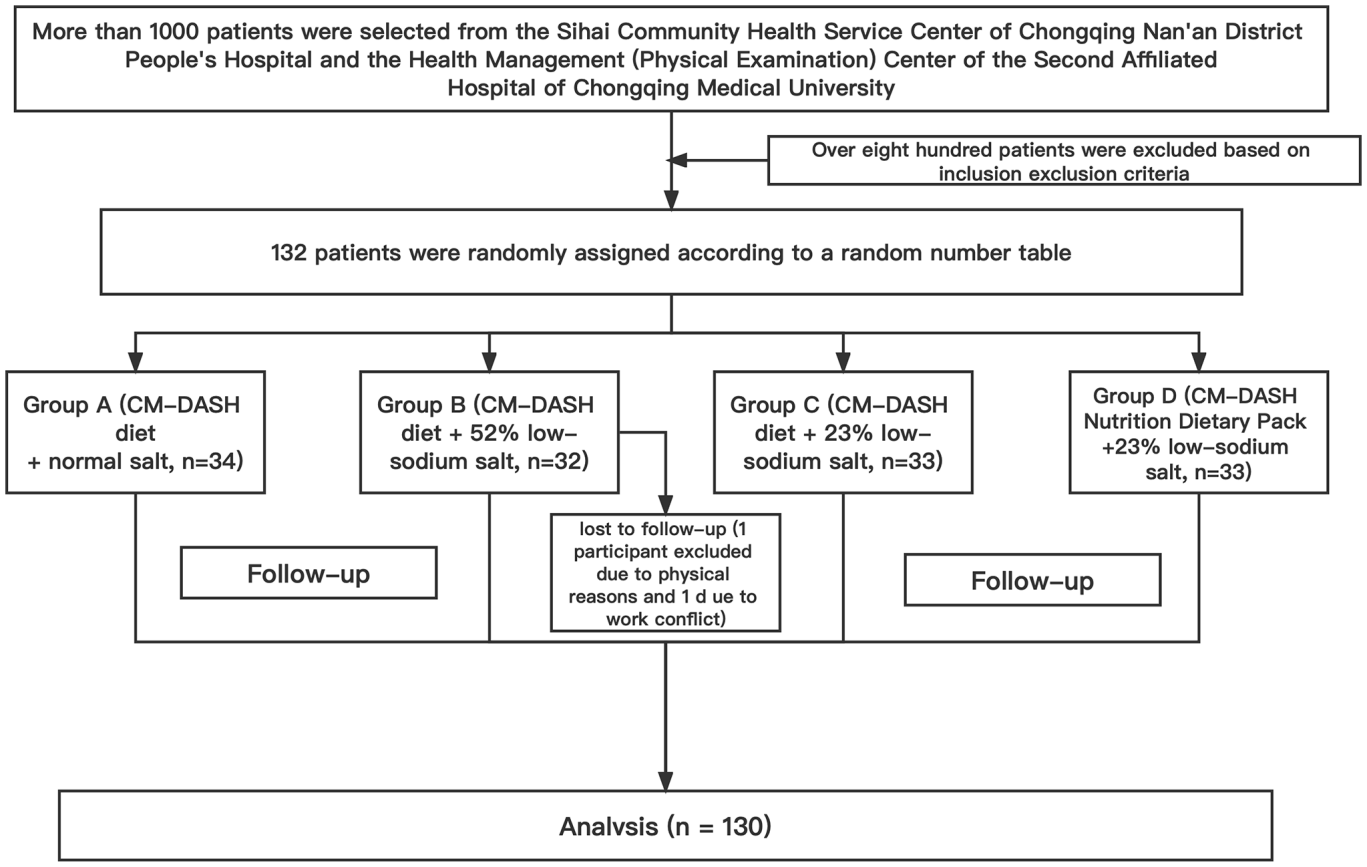
Flowchart of the study.

This trial was conducted in Chongqing, China, from July to December 2021; 132 participants who were eligible for the test were selected from the Sihai Community Health Service Center of Chongqing Nan’an District People’s Hospital and the Health Management (Physical Examination) Center of the Second Affiliated Hospital of Chongqing Medical University. The flow diagram depicting the enrolment of the participants in the study is shown in Figure 1.

#### 2.2.1. Inclusion criteria

The inclusion criteria were as follows: hypertension and type 2 diabetes; age ≥ 50 and ≤ 75 years; residence near the hospital without plan to move residence or leave the city during the trial; strict adherence to two daily meals provided by the investigators during the centralized feeding phase; and provision of informed consent (including their family members).

The diagnostic criteria for hypertension were based on the Chinese Guidelines for the Prevention and Treatment of Hypertension (2018 Revised Edition) (16), and the diagnostic criteria for diabetes were based on the Chinese Guidelines for the Prevention and Treatment of Type 2 Diabetes (2017 Edition) (17) (taking regular anti-hypertensive or hypoglycemic drugs).

#### 2.2.2. Exclusion criteria

The exclusion criteria were as follows: presence of malignant tumors, acute myocardial infarction, stroke in the past 3 months or other serious diseases and expected survival time < 1 year; increased cortisol disorder or aldosteronism; acute diseases, such as upper respiratory tract infection, fever, or severe diarrhea; disabilities, such as hearing or speech impairment, dementia, severe depression, or other mental disorders preventing normal communication; unavailability for regular follow-up or loss to follow-up after study entry; renal dysfunction or chronic kidney disease at stage 4 or above; abnormal liver function, with alanine aminotransferase or aspartate aminotransferase levels >2 times the upper limit of normal or total bilirubin level greater than the upper limit of normal; abnormal blood potassium level, <3.5 mmol/L or > 5.5 mmol/L, or current use of potassium-preserving diuretics; pregnancy, possibility of pregnancy, or other contraindications for the use of the trial meal; current consumption of a low-sodium diet or participation in other clinical studies; or other ineligible conditions as determined by the investigators.

### 2.3. Study design

We performed a randomized controlled single-blind trial (patients were blinded to the specific grouping) with a semi-open design. After completion of the baseline survey, physical examination, and 24-h urine collection, participants were randomly classified using a random number table method into Group A (CM-DASH diet + normal salt, *n* = 34); Group B (CM-DASH diet +52% low-sodium salt, *n* = 32); Group C (CM-DASH diet +23% low-sodium salt, *n* = 33); and Group D (CM-DASH Nutrition Dietary Pack +23% low-sodium salt, *n* = 33).

#### 2.3.1. Procedure

The intervention lasted for 8 weeks, of which the first and second weeks involved DASH diet adaptation at home (salt + CM-DASH diet), and the third and fourth weeks comprised the centralized feeding phase (centralized feeding in the hospital canteen). Breakfast was eaten at home according to the diet recommendations. Lunch and dinner were prepared by the hospital canteen staff according to the diet recommendations and salt requirements under the guidance and supervision of the researchers. Lunch was eaten in the hospital, dinner was eaten at home, and all patients were asked not to add extra salt. The fifth to eighth week comprised the home nursing stage (salt + CM-DASH diet). All patients participated in weekly follow-up visits wherein their blood pressure was measured and trial salt was distributed. Additionally, the participants were reminded to strictly abide by the requirements of salt intake and diet and to reduce the frequency of eating out. Specialized doctors were also required to monitor the participants’ drug use and ensure the safety of the trial.

#### 2.3.2. Salt use

The test salt was used throughout the trial as a substitute for the participants’ household salt. Group A received common salt (“Jing Xin,” manufactured by Chongqing Salt Industry Group Co., Ltd. Name: purified salt; product standard: NY/T1040; sodium chloride content: >99%). Group B received 52% low-sodium salt [“Gu Da Chu,” manufactured by Shanghai Institute of Ecological Health Sciences. Name: solid compound condiment; standard of execution: Q/BAAM0009S; food production license number: SC10334042205441; main ingredients included potassium chloride (31%), sodium chloride (52%), carbohydrates (9.2%), and protein (4.4%)]. Groups C and D received 23% low-sodium salt [“Shan Yi Kang,” manufactured by Chongqing Shanshun Biotechnology Co., Ltd. Name: solid compound condiment; standard of execution: Q/SWS 0025S; food production license number: SC10650012000709; main ingredients included potassium chloride (56%), sodium chloride (23%), and protein (3.0%)]. We provided a quantitative spoon for the participants to control the amount of salt used in the household (<5 g per person per day). In addition, we used an electronic scale (precision: 0.1 g) to weigh the salt consumption weekly and estimate the daily salt consumption for each family member.

#### 2.3.3. Chinese modified dietary approaches to stop hypertension recipes

According to the recommendations of the Chinese Guidelines for the Prevention and Control of Type 2 Diabetes (2018 Edition) and the Chinese Guidelines for the Prevention and Control of Hypertension (2017 Revision), respectively, patients with diabetes should adopt a diversified dietary pattern that is “based on cereals, high in dietary fiber intake, and low in salt, sugar, and fat,” and patients with hypertension should have a diet based on fruits, vegetables, low-fat dairy products, whole grains rich in dietary fiber, and plant-derived protein and should reduce the intake of saturated fat and cholesterol. Accordingly, the investigators combined the information on the DASH Dietary Energy Supply Estimation Table, the Dietary Pagoda for Chinese Residents (2016), the dietary habits of Chinese residents, the availability of food, as well as the participants’ body mass levels, incidence of obesity, and level of physical activity to create a diet suitable for Chinese patients with hypertension and type 2 diabetes mellitus. The CM-DASH dietary model was developed for Chinese patients with hypertension and type 2 diabetes based on the daily energy requirement per kilogram of ideal body mass for light physical activity in patients who are overweight or obese. The main requirements were as follows: (1) use of brown rice, corn, soybeans, red beans and other grains to make mixed-grain rice; (2) use of lean meat and poultry, an average daily intake of lean meat, poultry, fish, and other meats of 80–120 g and minimization of consumption of red meat and eating more white meat (skinless chicken and fish); (3) daily consumption of approximately 250 g of low-fat milk and dairy products (high calcium); (4) Eating more low-sugar green leafy vegetables (about 500 g/day); (5) ensuring a moderate intake of fruit (after meals) and nuts (one small handful); and (6) a daily intake of edible oil (vegetable oil) <30 g, and salt intake <5 g. The CM-DASH diet contains less fat, saturated fat, and calories than the standard DASH diet and is more in line with Chinese dietary habits. An older man with hypertension and type 2 diabetes mellitus with a body mass of about 60 kg who engaged in light physical activity was used as an example. All participants cooked food at home (during the first and second, and fifth to eighth weeks) using the recommended recipes (Table 1).

**Table 1:** Nutrient composition of CM-DASH (Chinese Modified Dietary Approaches to Stop Hypertension) diet.

Food		Net mass (g)	Energy (kcal)	Protein (g)	Fat (g)	Carbohydrates (g)	Dietary fiber(g)	Sodium (mg)	Potassium (mg)	Magnesium (mg)	Calcium (mg)
Breakfast	Whole-wheat bread	100.00	163.00	10.50	0	52.30	4.20	265.00	250.00	77.00	163.00
	Low-fat, high-calcium milk	250.00	110.00	7.50	3.25	12.25	0	180.00	272.50	27.50	312.50
	Egg	50.00	71.50	6.05	5.25	0.05	0	65.00	65.00	0	17.50
Lunch	Mixed grain rice	60.00	69.00	1.93	0.43	18.24	0.28	0.53	34.61	11.42	3.91
	Meat (chicken, fish)	60.00	59.20	12.09	1.11	0.18	0	38.04	213.90	17.70	0.60
	Vegetables (dark green vegetables)	250.00	46.25	3.19	0.56	10.19	4.13	38.75	463.13	43.75	60.00
Dinner	Mixed grain rice	60.00	69.00	1.93	0.43	18.24	0.28	0.53	34.61	11.42	3.91
	Meat (chicken, fish)	60.00	59.20	12.09	1.11	0.18	0	38.04	213.90	17.70	0.60
	Vegetables (dark green vegetables)	250.00	46.25	3.19	0.56	10.19	4.13	38.75	463.13	43.75	60.00
Others	Nut (walnut)	20.00	129.20	2.98	11.76	3.82	1.90	1.28	77.00	26.20	11.20
	Fruit	150.00	79.50	0.60	0.30	20.55	2.55	1.95	124.50	6.00	6.00
	Cooking oil	20.00	179.80	0	19.98	0	0	0.70	0.20	0.40	2.40
	Salt	5.00	48.77	0.22	0	0.46	0	1025.30	807.75	2.00	22.00
Total		1335.00	1130.67	62.27	44.74	146.65	17.46	1693.87	3020.23	284.84	663.62
Sample of the menus		Breakfast				Lunch			Dinner		
		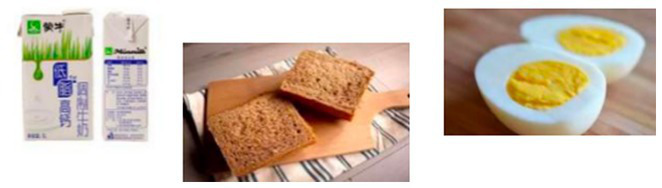				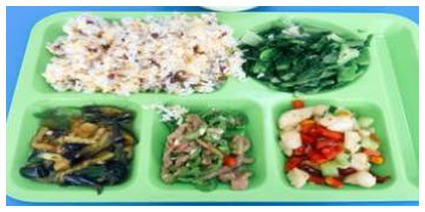			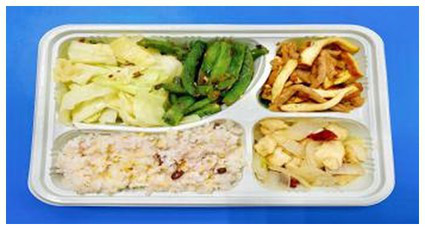		

#### 2.3.4. Meal packs

The meal packs (CM-DASH Nutrition Dietary Pack, manufactured by Chongqing Shanshun Biotechnology Co., Ltd. Product standards: GB/T29603; food production license No. SC12450023228393) dietary treatment for type 2 diabetes and hypertension is based on Chinese dietary structure and habits; it follows the principles of dietary treatment of hypertension and diabetes, screening the use of Chinese medicines in the treatment of hypertension and diabetes and the results of using modern hypertension and diabetes nutritional medicine. It uses mixed-grain and homogenized meals as the core of the nutritional intervention treatment and was provided as an intervention for Group D. It included the following items: CM-DASH Homogenized Meal Solid Drink (homogenized meal for breakfast), CM-DASH Solid Drink (Normal Companion) and CM-DASH Eight Treasures Rice (Rice for Mid-Dinner). The ingredients of each item are shown in Table 2.

**Table 2:** The meal pack (CM-DASH Nutrition Dietary Pack) composition.

Nutrient content	CM-DASH Homogenized Meal Solid Drink (Homogenized Meal for Breakfast)	CM-DASH Solid Drink (Normal Companion)	CM-DASH Eight Treasures Rice (Rice for Mid-Dinner)
Weight (g)	39	60	200
Energy (kcal)	119.74	171.36	722.26
Protein (g)	8.4	8.8	38.4
Fat (g)	1.5	2.7	14.4
Carbohydrates (g)	17.7	27.5	118.8
Fiber (g)	—	—	14.97
Sodium (mg)	90	26	10
Potassium (mg)	—	—	—
Magnesium (mg)	60	—	—
Calcium (mg)	120	—	—
Sample	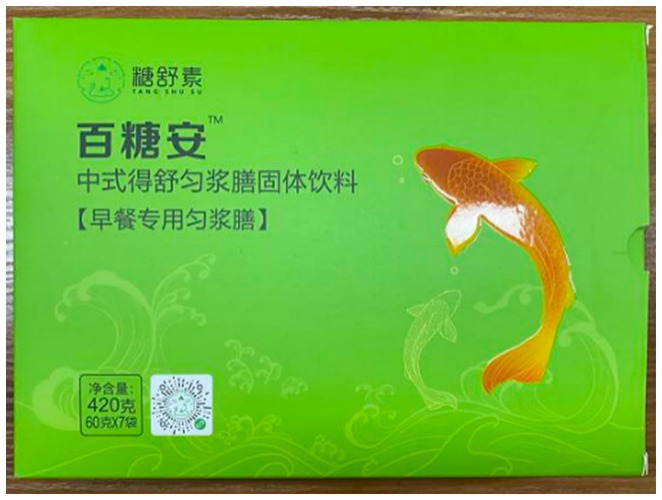	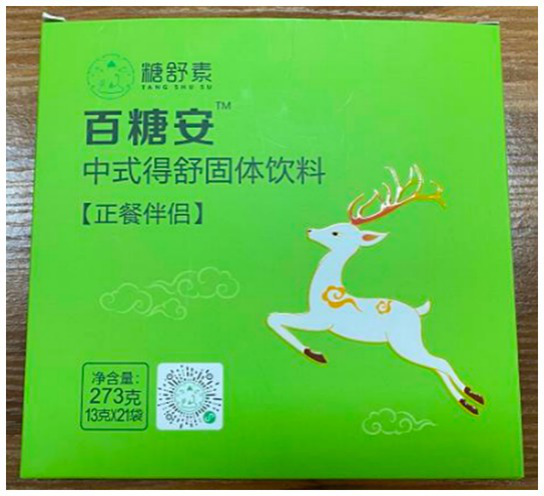	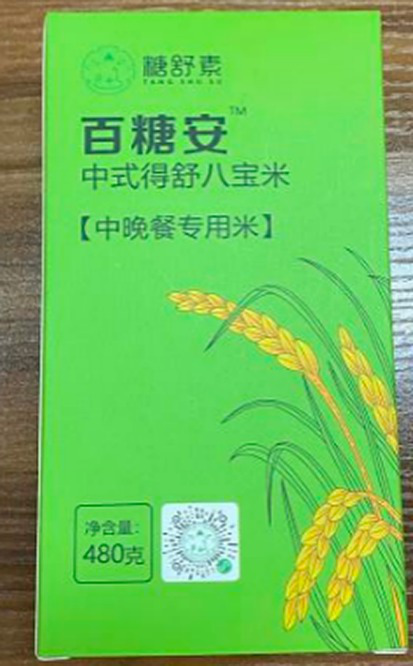

### 2.4. Blood pressure measurement

Participants attended follow-up appointments in the hospital once a week. Blood pressure was measured three times by trained professionals between 8 and 9 a.m., with an interval of about 2 min. The average of the last two blood pressure measurements was used for the analysis. According to the oscillometer technique, automated upper-arm cuff devices were used (Omron, Dalian, Co., Ltd. Product name: “Omron” electronic sphygmomanometer; model: HEM-7130).

### 2.5. Laboratory measurements

Laboratory measurements included 24-h urinary electrolytes and blood electrolytes. All indices were tested in the Second Affiliated Hospital of Chongqing Medical University. The test instrument was a fully automated chemiluminescence immunoassay analyzer (Model: Cobas 6,000 e 601; manufacturer: Roche Diagnostics GmbH).

### 2.6. Statistical analysis

The normality of the data was tested using the Kolmogorov–Smirnov test, with normally distributed data expressed as mean ± standard deviation (M ± SD) and non-normally distributed data expressed as median and upper and lower quartiles (P25, P75). Levene’s test was used to test the data’s homogeneity of variance. Inter-and intra-group comparisons were performed using the paired t-test and one-way analysis of variance, as well as the Kruskal–Wallis H test and Wilcoxon’s signed-rank test. The blood pressure values measured continuously were analyzed using a generalized estimation equation (GEE). SPSS version 26.0 (IBM, Armonk, NY, United States) was used for all statistical analyzes. All tests were two-sided, and *p* < 0.05 was considered statistically significant.

### 2.7. Ethics approval and consent to participate

The study protocol was approved by the Ethics Committee of Chongqing Medical University (approval date: 07/28/2020). All procedures reported in this study have been conducted in an ethical and responsible manner and in full compliance with all relevant codes of experimentation and legislation (Trial registration: ChiCTR2000029017. Registered January 11, 2020-Prospective registration, http://www.chictr.org.cn/). All participants and their families provided written informed consent before enrollment in the trial.

## 3. Results

### 3.1. Demographic characteristics

In total, 132 participants were included in the study, and two were lost to follow-up, with a 1.5% loss rate; therefore, 130 participants completed the trial. There were no significant differences in demographic characteristics of the participants in the four groups except for age (Table 3).

**Table 3:** Baseline of participants.

Characteristics	Group A (control group, *n* = 34)	Group B (52% low-sodium salt group, *n* = 30)	Group C (23% low-sodium salt group, *n* = 33)	Group D (meal pack group, *n* = 33)	*p*
Gender (Male, %)	16 (47.1)	11 (36.7)	17 (51.5)	16 (48.5)	0.670^a^
Age (y)	68.12 ± 3.95	70.00 ± 4.24	63.39 ± 6.83	67.21 ± 5.19	0.000^b*^
BMI (kg/m^2^)	24.96 ± 2.12	25.34 ± 3.09	25.46 ± 8.66	24.97 ± 4.42	0.719^b^
Waist circumference (cm)	84.41 ± 6.65	84.80 ± 8.47	86.67 ± 8.06	86.36 ± 11.12	0.654^b^
Hip circumference (cm)	92.03 ± 5.56	92.13 ± 5.60	95.21 ± 7.29	95.06 ± 8.56	0.122^b^
Waist-to-hip ratio	0.92 ± 0.05	0.92 ± 0.01	0.90 ± 0.06	0.91 ± 0.06	0.623^b^
Body fat percentage (%)	31.30 ± 5.92	30.93 ± 6.91	30.30 ± 5.24	30.77 ± 6.55	0.929^a^
SBP (mmHg)	136.15 ± 15.39	139.72 ± 15.89	133.00 ± 14.48	135.70 ± 13.71	0.363^b^
DBP (mmHg)	79.81 ± 7.90	79.07 ± 8.89	77.97 ± 7.48	77.42 ± 8.27	0.630^b^
Years of hypertension (y)	11.28 ± 6.66	9.38 ± 7.11	10.23 ± 7.19	12.53 ± 9.06	0.513^b^
Years of diabetes (y)	8.18 ± 6.04	10.40 ± 7.61	10.56 ± 8.40	12.27 ± 7.67	0.179^b^
Family history of hypertension (*n*, %)	18 (52.94)	14 (46.67)	15 (45.45)	22 (66.67)	0.094^a^
Family history of diabetes (*n*, %)	12 (35.3)	11 (36.7)	13 (39.40)	15 (45.45)	0.714^a^
Drinking (*n*, %)	6 (17.65)	3 (10.0)	5 (15.15)	6 (18.18)	0.801^a^
Smoking (*n*, %)	3 (8.8)	1 (3.3)	4 (12.1)	2 (6.1)	0.594^c^
Using statins (*n*, %)	14 (41.2)	8 (26.7)	10 (30.3)	17 (51.5)	0.159^a^
Using aspirin (*n*, %)	8 (23.5)	5 (16.7)	6 (18.2)	8 (24.2)	0.840^a^
ACEI (*n*, %)	3 (10.0)	0 (0.0)	1 (3.03)	1 (3.03)	0.336^c^
Diuretics (*n*, %)	5 (16.7)	4 (12.12)	7 (21.21)	4 (12.12)	0.754^a^
ARB (*n*, %)	14 (41.18)	12 (40.0)	19 (57.58)	17 (51.52)	0.202^a^
CCB (*n*, %)	19 (55.88)	19 (63.33)	15 (45.45)	12 (36.36)	0.214^a^
β-blockers (*n*, %)	6 (25.0)	1 (3.3)	4 (12.12)	4 (12.12)	0.385^a^

^a^R*C Chi-square test for contingency tables. ^b^one-way ANOVA. ^c^Fisher’s precision probability test.

*There was difference between Group C and Group A (*p* = 0.031), and there was difference between Group C and Group B (*p* = 0.000).

### 3.2. Blood pressure changes

**Figure 2:**
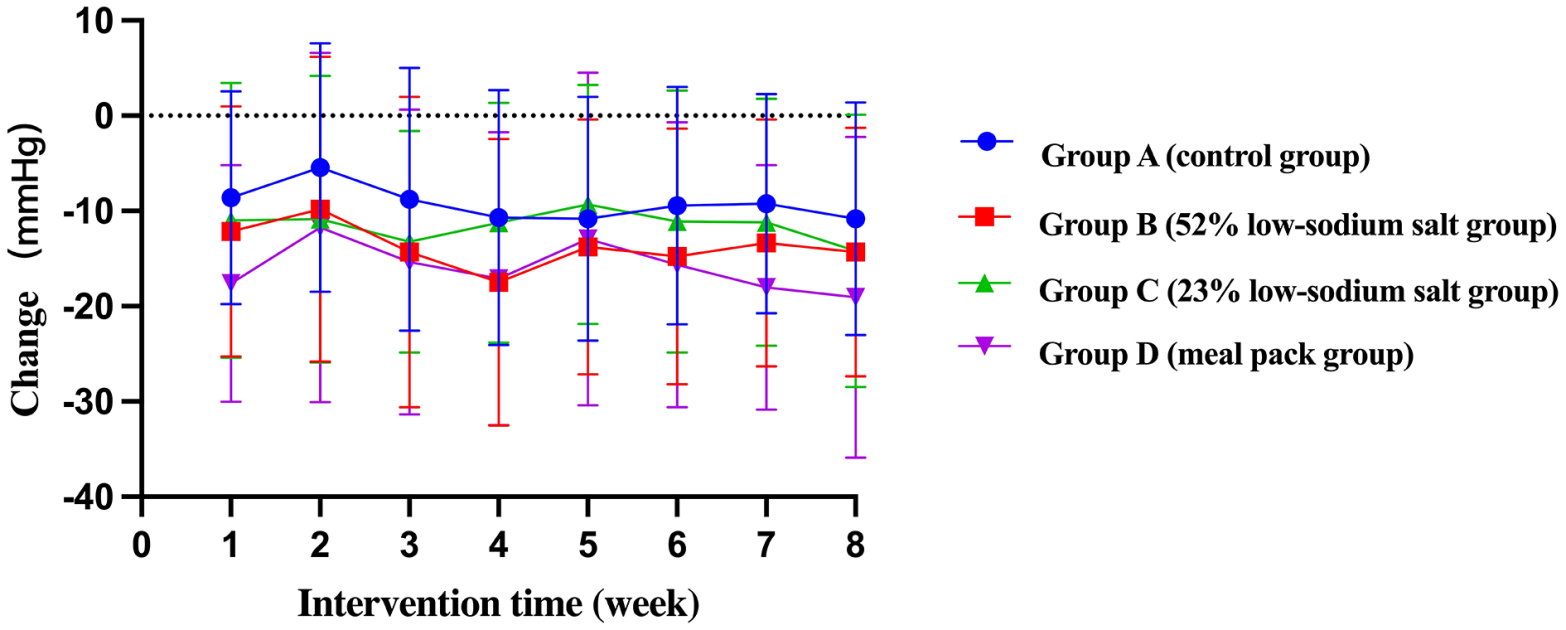
Weekly SBP change trend of patients.

**Figure 3:**
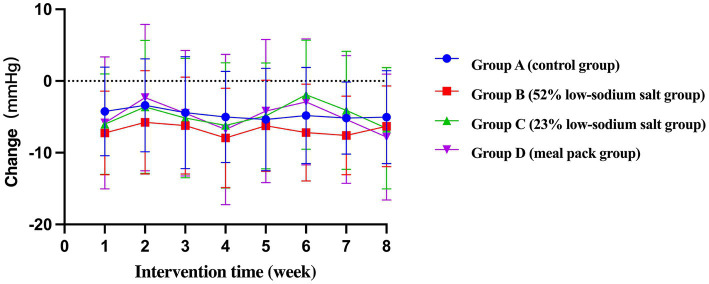
Weekly DBP change trend of patients.

Compared to baseline, the mean systolic blood pressures (SBPs) of Groups C and D were lower than those of Group A [−6.54 mmHg, 95% confidence interval (CI) -12.58 to −0.49, *p* = 0.034; −8.70 mmHg, 95% CI -14.75 to −2.66, *p* = 0.005] and Group B (−6.60 mmHg, 95% CI -12.84 to −0.36, *p* = 0.038; −8.76 mmHg, 95% CI -15.00 to −2.52, *p* = 0.006). The mean diastolic blood pressure (DBP) of Group D was lower than that of Group A (−5.17 mmHg, 95% CI -8.86 to −1.48, *p* = 0.006). The GEE showed a significant decrease in blood pressure from baseline in all groups, with a more significant decrease in Group D compared with all other groups (SBP: −19.06 mmHg, 95% CI -24.71 to −13.41, *p* < 0.001 and DBP: −7.82 mmHg, 95% CI -10.77 to −6.87, *p* < 0.001; Tables 4, 5; Figures 2, 3).

**Table 4:** Changes in SBP from baseline during intervention.

	Group A (control group, *n* = 34)			Group B (52% low-sodium salt group, *n* = 30)			Group C (23% low-sodium salt group, *n* = 33)			Group D (meal pack group, *n* = 33)			p**
	SBP(mmHg, M ± SD)	Change(MD, 95% CI)	p*	SBP (mmHg, M ± SD)	Change (MD, 95% CI)	p*	SBP (mmHg, M ± SD)	Change (MD, 95% CI)	p*	SBP (mmHg, M ± SD)	Change (MD, 95% CI)	p*	
Baseline	136.15 ± 15.39	—	—	139.72 ± 15.98	—	—	133.00 ± 14.48	—	—	135.70 ± 13.71	—	—	0.363
1w	127.53 ± 13.26	−8.62 (−12.32, -4.92)	<0.001	127.55 ± 10.49	−12.17 (−16.80, -7.54)	<0.001	122.01 ± 11.46	−10.99 (−15.83, -6.15)	<0.001	118.09 ± 9.39	−17.61 (−21.78, -13.43)	<0.001	0.001^a^
2w	130.71 ± 15.21	−5.44 (−9.77, -1.12)	0.014	129.88 ± 11.56	−9.83 (−15.48, -4.19)	0.001	122.14 ± 14.94	−10.86 (−15.92, -5.81)	<0.001	123.95 ± 15.34	−11.75 (−17.92, -5.58)	<0.001	0.039^b^
3w	127.37 ± 13.93	−8.78 (−13.35, -4.21)	<0.001	125.38 ± 14.35	−14.33 (−20.07, -8.60)	<0.001	119.79 ± 12.32	−13.21 (−17.12, -9.30)	<0.001	120.33 ± 11.92	−15.36 (−20.74, -9.99)	<0.001	0.050^c^
4w	125.46 ± 13.12	−10.69 (−15.12, -6.26)	<0.001	122.23 ± 11.67	−17.48 (−22.78, -12.18)	<0.001	123.79 ± 11.91	−11.21 (−15.45, -6.98)	<0.001	118.59 ± 12.40	−17.11 (−22.27, -11.94)	<0.001	0.161
5w	125.32 ± 11.17	−10.82 (−15.07, -6.58)	<0.001	125.95 ± 8.60	−13.77 (−18.48, -9.06)	<0.001	123.70 ± 9.60	−9.30 (−13.51, -5.09)	<0.001	122.77 ± 13.82	−12.92 (−18.79, -7.06)	<0.001	0.642
6w	126.72 ± 9.84	−9.43 (−13.56, -2.91)	<0.001	124.96 ± 11.36	−14.75 (−19.48, -10.03)	<0.001	121.88 ± 12.82	−11.12 (−15.75, -6.49)	<0.001	120.05 ± 12.58	−15.65 (−20.68, -10.62)	<0.001	0.096
7w	126.93 ± 10.49	−9.22 (−13.03, -5.41)	<0.001	126.35 ± 10.82	−13.37 (−17.94, -8.80)	<0.001	121.80 ± 12.34	−11.20 (−15.55, -6.85)	<0.001	117.68 ± 11.18	−18.02 (−22.33, -13.70)	<0.001	0.003^d^
8w	125.34 ± 12.29	−10.81 (−14.85, -6.77)	<0.001	125.40 ± 9.96	−14.32 (−18.91, -9.73)	<0.001	118.80 ± 12.28	−14.20 (−19.01, -9.39)	<0.001	116.64 ± 14.78	−19.06 (−24.71, -13.41)	<0.001	0.007^e^

*The GEE was used for analysis, adjusting gender, age, smoking, drinking, family history of hypertension, age of hypertension, waist-to-hip ratio, baseline BMI and statins, aspirin use. **Comparison between groups.

^a^Group C vs. Group A, −5.52 (−10.97 ~ −0.07) mmHg, *p* = 0.047; Group D vs. Group A, −9.44 (−14.89 ~ −3.99) mmHg, *p* = 0.001; Group D vs. Group B, −9.46 (−15.09 ~ −3.83) mmHg, *p* = 0.001. ^b^Group C vs. Group A, −8.57 (−15.54 ~ −1.60) mmHg, *p* = 0.016; Group C vs. Group B, −7.75 (−14.94 ~ −0.55) mmHg, *p* = 0.035. ^c^Group C vs. Group A, −7.58 (−13.94 ~ −1.22) mmHg, *p* = 0.020; Group D vs. Group A, −7.03 (−13.39 ~ −0.68) mmHg, *p* = 0.030. ^d^Group D vs. Group A, −9.24 (−14.68 ~ −3.81) mmHg, *p* = 0.001; Group D vs. Group B, −8.67 (−14.28 ~ −3.06) mmHg, *p* = 0.003. *e*: Group C vs. Group A, −6.54 (−12.58 ~ −0.49) mmHg, *p* = 0.034; Group D vs. Group A, −8.70 (−14.75 ~ −2.66) mmHg, *p* = 0.005; Group C vs. Group B, −6.60 (−12.84 ~ −0.36), *p* = 0.038; Group D vs. Group B, −8.76 (−15.00 ~ −-2.52) mmHg, *p* = 0.006.

**Table 5:** Changes in DBP from baseline during intervention.

	Group A (control group, *n* = 34)			Group B (52% low-sodium salt group, *n* = 30)			Group C (23% low-sodium salt group, *n* = 33)			Group D (meal pack group, *n* = 33)			p**
	DBP(mmHg, M ± SD)	Change(MD, 95% CI)	p*	DBP(mmHg, M ± SD)	Change(MD, 95% CI)	p*	DBP(mmHg, M ± SD)	Change(MD, 95% CI)	p*	DBP(mmHg, M ± SD)	Change(MD, 95% CI)	p*	
Baseline	79.81 ± 7.90	—	—	79.07 ± 8.89	—	—	77.97 ± 7.48	—	—	77.42 ± 8.27	—	—	0.630
1w	75.56 ± 8.86	−4.25 (−6.30, -2.20)	<0.001	71.82 ± 8.77	−7.25 (−9.31, -5.19)	<0.001	71.95 ± 7.73	−6.02 (−8.38, -3.67)	<0.001	71.58 ± 7.44	−5.85 (−8.94, -2.76)	<0.001	0.154
2w	76.43 ± 9.55	−3.38 (−5.53, -1.24)	0.002	73.30 ± 9.52	−5.77 (−8.30, -3.23)	<0.001	74.36 ± 8.78	−3.61 (−6.73, -0.50)	0.023	75.12 ± 7.39	−2.30 (−5.73, 1.13)	0.188	0.546
3w	75.40 ± 8.36	−4.41 (−6.99, -1.83)	0.001	72.85 ± 9.98	−6.22 (−8.60, -3.83)	<0.001	72.82 ± 9.08	−5.15 (−7.95, -2.35)	<0.001	72.92 ± 7.27	−4.50 (−7.44, -1.56)	0.003	0.547
4w	74.79 ± 7.12	−5.02 (−7.12, -2.91)	<0.001	71.12 ± 8.76	−7.95 (−10.39, -5.51)	<0.001	71.79 ± 9.01	−6.18 (−9.12, -3.25)	<0.001	70.67 ± 8.31	−6.76 (−10.28, -3.23)	<0.001	0.176
5w	74.44 ± 7.69	−5.37 (−7.73, -3.01)	<0.001	71.83 ± 7.98	−6.24 (−8.47, -4.01)	<0.001	73.11 ± 9.38	−4.86 (−7.34, -2.39)	<0.001	73.24 ± 9.75	−4.18 (−7.53, -0.83)	0.014	0.883
6w	74.99 ± 7.14	−4.82 (−7.04, -2.60)	<0.001	71.88 ± 8.67	−7.18 (−9.56, -4.81)	<0.001	76.05 ± 8.20	−1.92 (−4.48, 0.63)	0.140	74.52 ± 7.71	−2.91 (−5.86, 0.04)	0.054	0.206
7w	74.65 ± 6.79	−5.16 (−6.83, -3.50)	<0.001	71.47 ± 8.32	−7.60 (−9.53, -5.67)	<0.001	73.89 ± 8.58	−4.08 (−6.84, -1.32)	0.004	72.06 ± 7.79	−5.36 (−8.35, -2.37)	<0.001	0.325
8w	74.78 ± 6.59	−5.03 (−7.17, -2.88)	<0.001	72.75 ± 7.43	−6.32 (−8.30, -4.33)	<0.001	71.38 ± 8.83	−6.59 (−9.43, -3.75)	<0.001	69.61 ± 7.51	−7.82 (−10.77, -6.87)	<0.001	0.046^a^

*The GEE was used for analysis, adjusting gender, age, smoking, drinking, family history of hypertension, age of hypertension, waist-to-hip ratio, baseline BMI and statins, aspirin use. **Comparison between groups.

^a^Group D vs. Group A, −5.17 (−1.48 ~ −8.86) mmHg, *p* = 0.006.

### 3.3. Blood and urine biochemical changes

At the end of the intervention, the 24-h urine Na^+^/K^+^ ratio decreased in the low-sodium group (p < 0.001). Except for Group A, all of the groups showed a trend of increase in potassium and decrease in sodium 24-h after the intervention, with no difference among groups. As an indicator of safety, serum K^+^ and Na^+^ fluctuated during the trial but were within normal limits (Table 6).

**Table 6:** Biomarker parameters.

	Group A (control group, *n* = 34)				Group B (52% low-sodium salt group, *n* = 30)				Group C (23% low-sodium salt group, *n* = 33)				Group D(meal pack group, n = 33)				p1*	p2**	p3***
	Baseline	4th week	After	*p*	Baseline	4th week	After	*p*	Baseline	4th week	After	*p*	Baseline	4th week	After	*p*			
serum K^+^(mmol/h)	4.15 ± 0.40	4.71 ± 0.47	4.65 ± 1.90	0.000	3.96 ± 0.62	4.92 ± 0.27	4.31 ± 0.36	0.000	4.13 ± 0.31	4.33 ± 0.29	4.35 ± 0.72	0.059	4.20 ± 0.31	4.49 ± 0.50	4.40 ± 0.37	0.013	0.199	0.000	0.702
serum Na^+^(mmol/h)	142.42 ± 2.24	141.78 ± 2.10	138.87 ± 1.64	0.280	141.84 ± 1.51	140.89 ± 1.19	140.38 ± 2.28	0.015	140.63 ± 1.42	142.12 ± 1.54	142.81 ± 1.76	0.000	140.19 ± 2.01	140.67 ± 1.77	142.77 ± 1.97	0.000	0.000	0.008	0.000
24 h U K^+^(mmol/24 h)	49.54 ± 24.11	44.08 ± 13.32	45.01 ± 19.57	0.259	43.75 ± 18.27	44.37 ± 16.07	55.26 ± 24.76	0.000	45.28 ± 22.67	50.84 ± 17.19	55.80 ± 21.07	0.334	42.97 ± 23.07	71.25 ± 25.11	57.41 ± 20.21	0.000	0.694	0.000	0.082
24 h U Na^+^(mmol/24 h)	143.70 ± 58.34	121.43 ± 41.07	129.13 ± 54.17	0.078	153.95 ± 43.01	104.66 ± 30.96	130.54 ± 52.97	0.000	155.21 ± 58.55	131.51 ± 45.71	132.15 ± 64.84	0.042	147.43 ± 74.44	121.95 ± 52.15	131.36 ± 67.50	0.070	0.085	0.115	0.998
24 h U Na^+/^K^+^	3.29 ± 1.53	2.85 ± 0.97	3.09 ± 1.23	0.127	4.07 ± 1.98	2.53 ± 0.87	2.67 ± 1.40	0.000	3.93 ± 1.56	2.77 ± 1.11	2.47 ± 0.98	0.000	3.62 ± 1.51	1.85 + 0.78	2.36 ± 1.05	0.000	0.072	0.926	0.424

*Baseline comparison between groups. **Week 4 comparison between groups. ***Comparison between groups after intervention.

### 3.4. Adverse events

No serious adverse reactions occurred during the intervention. Mild symptoms, such as dizziness, fatigue, diarrhea, abdominal pain were observed in 10 cases (7.69%) during the follow-up period, of which two cases (5.88%) were in Group A, two cases (6.67%) were in Group B, one case (3.03%) was in Group C, and five cases (15.15%) were in Group D, three of which were hypoglycemic adverse reactions. All adverse reactions were short-term symptoms that resolved on their own (Table 7).

**Table 7:** Adverse events.

No.	Group	Symptoms	Time (Week)	Measures	Transfer to
120,206	A	Blurred eyes, Regurgitation	1	-	Recovery
121,007	A	Diarrhea	3	-	Recovery
121,101	B	Diarrhea	3	Symptomatic treatment	Recovery
100,910	B	Diarrhea	5	-	Recovery
092807	C	Lack of power	2	Use of common salt	Recovery
092737	D	Indigestion	2	Take medication (Morpholine)	Recovery
092834	D	Hypoglycemia	2	Eating cookies	Recovery
092832	D	Hypoglycemia	2	Eating cookies	Recovery
092711	D	Hypoglycemia	2	Reduce glucose-lowering medication as prescribed by doctor	Recovery
092710	D	Diarrhea	3	Take medication (Norfloxacin)	Recovery

## 4. Discussion

Lifestyle interventions, especially dietary interventions, are essential for the prevention and management of chronic diseases such as hypertension and diabetes. Among the many dietary patterns worldwide, the Eastern (more vegetables, fruits, and low saturated fat) and Mediterranean dietary patterns have been shown to have positive effects (4, 18–20), whereas the Western dietary pattern (predominant intake of red meat, processed meat, high saturated fat, and low dietary fiber) exacerbates the risks of hypertension and diabetes (21, 22). Existing dietary patterns are difficult to apply in other regions due to limited ingredients and eating culture; therefore, some easily accessible dietary patterns are gradually proposed. A clinical trial of 459 patients with hypertension showed that the DASH diet decreased the SBP and DBP by 5.5 mmHg and 3.0 mmHg, respectively, compared to regular diet (23). Another meta-analysis revealed that the DASH diet significantly reduced the SBP (mean 6.74 mmHg) and DBP (mean 3.54 mmHg) in patients with hypertension (24). In the DASH diet, the daily sodium intake should be <2,300 mg (equivalent to 5.8 g of salt with >99% NaCl) (25), which is difficult to achieve without changing salt-use habits. Thus, some studies have explored combining the DASH diet with low-sodium salt. However, to our knowledge, the use of 52 and 23% low-sodium salts in combination with a modified DASH dietary pattern with severely restricted ingredients to assess their effects on blood pressure in patients with hypertension and type 2 diabetes has not yet been reported in China.

The results of this study showed that patients with hypertension and type 2 diabetes had a significant decrease in SBP/DBP immediately after the start of CM-DASH dietary intervention. At the end of the first week of intervention, the mean decreases were 8.62 mmHg/4.25 mmHg, 12.17 mmHg/7.25 mmHg, 10.99 mmHg/ 6.02 mmHg, and 17.61 mmHg/ 5.85 mmHg in Groups A–D, respectively. The decrease in SBP was most pronounced in Group D, which could be because the components of the meal pack (CM-DASH Nutrition Dietary Pack) included 23% low-sodium salt and various supplementary foods rich in dietary fiber (lunch/dinner staple: 100 g CM-DASH Babao rice contain 7.486 g dietary fiber, whereas 45 g rice +10 g corn grits +10 g red beans only contain 1.5 g dietary fiber). The SBP of Group C was not significantly lower than that of Group B, possibly because the 23% low-sodium salt had a lower sodium content and a more unpalatable taste than the 52% low-sodium salt and common salt; the patients were in the stage of adaptation to low-salt diet in the first week. However, the results consistently showed that the decrease in blood pressure was significant after the patients changed their unbalanced dietary habits (e.g., high-salt diet). Even in the absence of NaCl restriction (Group A), salt intake restriction combined with a modified DASH diet resulted in a decrease in blood pressure, with implications for shortening the duration of their treatment and saving health care resources.

During the centralized feeding period (weeks 3 and 4), blood pressure decreased below baseline in all four groups (−10.69/−5.02 mmHg, −17.48/−7.95 mmHg, −11.21/−6.18 mmHg, −17.11/−6.76 mmHg) and remained at these levels, though there was a small increase in the later period. This result indicates that the centralized feeding model has good health benefits and is a practical guide for controlling blood pressure. At the end of the intervention, the blood pressure of Groups A–D decreased by 10.81 mmHg/5.03 mmHg, 14.32 mmHg/6.32 mmHg, 14.20 mmHg/ 6.59 mmHg, and 19.06 mmHg/ 7.82 mmHg, respectively. The results of our study corroborate those of previous studies wherein the decrease in SBP was significantly greater than that in DBP; SBP is an important reference index for predicting cardiovascular disease risk factors among older patients with hypertension. Thus, an SBP reduction can effectively lower the risk for future cardiovascular and cerebrovascular adverse events (26–28). The four groups did not show a significant difference in the short-term blood pressure lowering effect. Further study with a larger sample size is needed to observe changes in blood pressure in the patient population.

A meta-analysis examining the relationship between salt intake and blood pressure showed that every 1.0 g/day reduction in salt intake in patients with hypertension resulted in a 1.2 mmHg decrease in SBP (29). The results of a survey in northern China also showed that 58.6% of patients with hypertension were salt-sensitive (30), and when salt intake was reduced in this group, blood pressure could be effectively controlled. In this trial, the daily sodium intake of patients in Group B was only about half of that in Group A, and the daily sodium intakes in Groups C and D were only approximately a fifth of that in Group A. Since 90% of dietary salt is excreted through the kidneys, 24-h urinary sodium level remains the most accurate estimate of dietary salt intake (31). Sodium intake was estimated by 24-h urinary sodium testing, and urinary sodium excretion was positively correlated with SBP and DBP. The 24-h urinary sodium and potassium levels improved in all four groups after the intervention. The 24-h urinary sodium of Groups A–D decreased by 0.5, 1.1, 0.6, and 0.5 g, respectively, from baseline during the first phase of the intervention (weeks 1–4) and by 0.3, 0.5, 0.6, and 0.3 g, respectively, from baseline at the end of the intervention. During the first phase of the intervention (weeks 1–4), the 24-h urinary potassium levels of Groups C and D increased by 0.2 and 1.1 g from the baseline, respectively, with a statistically significant difference among the four groups (*p* = 0.000). At the end of the intervention, the respective 24-h urinary potassium levels of Groups B, C, and D increased by 0.4, 0.4, and 0.7 g, respectively, from the baseline. Overall, our results showed that, even short-term lifestyle (dietary) interventions can yield good health benefits. We speculate that a more pronounced improvement in sodium and potassium intakes in an extended intervention period would result in even greater benefits to the prognosis of patients with hypertension and type 2 diabetes.

Furthermore, a previous study has shown that the urine Na^+^/K^+^ ratio can better predict sodium and potassium content in the body (32). The 24-h urine Na^+^/K^+^ ratios in the groups receiving low-sodium salt were significantly lower after the intervention than before it, indicating that low-sodium salt can effectively reduce the dietary sodium content while also supplementing a certain amount of potassium that helps to improve blood pressure levels. Serum test results showed a slight increase in potassium during the intervention period; however, it remained within the normal range. Due to the semi-open experimental design of this trial, confounding factors, such as interference and contamination, were inevitably encountered during the process. Moreover, the sample size of the trial was relatively small, and the short-term intervention of 8 weeks may not have resulted in a complete salt-reduction effect.

The long-term nature of chronic diseases poses a major challenge in healthcare systems worldwide. Patients who suffer from chronic diseases will remain in poor health and require medical care for long periods (33). To reduce the burden of chronic diseases and manage the pressure this situation may place on health systems, a series of well-designed, sustained, and complementary preventive and curative interventions are needed. Our study preliminarily shows that dietary therapy is safe and feasible and, if combined with comprehensive measures such as nutrition education, can improve patients’ health. We suggest that for specific populations, such as patients with refractory hypertension, the meal packs (CM-DASH Nutrition Dietary Pack) may be used in combination with low-sodium salt as a therapeutic diet to achieve better outcomes.

## 5. Conclusion

Our preliminary findings suggest that an 8-week intervention using 23 and 52% concentrations of low-sodium salt combined with a CM-DASH diet can achieve “salt reduction to scale” and thus safely improve sodium and potassium intake for blood pressure control in patients with hypertension and type 2 diabetes. Salt reduction as performed in this study is safe, has good patient compliance, and has practical value for dissemination to specific populations, such as patients with hypertension and type 2 diabetes.

## Data availability statement

The raw data supporting the conclusions of this article will be made available by the authors, without undue reservation.

## Ethics statement

The studies involving humans were approved by Ethics Committee of Chongqing Medical University (approval date: 07/28/2020). The studies were conducted in accordance with the local legislation and institutional requirements. The participants provided their written informed consent to participate in this study.

## Author contributions

LM contributed to the conception and design of the study. All authors contributed to the article and approved the submitted version.

## Funding

This research was supported by the Science and Technology Bureau of Chongqing (STBC), China (Project No: cstc2019jscx-msxm X0267). However, STBC did not participate in the design, implementation, data collection, analysis, or manuscript writing processes of the study.

## Conflict of interest

The authors declare that the research was conducted in the absence of any commercial or financial relationships that could be construed as a potential conflict of interest.

## Publisher’s note

All claims expressed in this article are solely those of the authors and do not necessarily represent those of their affiliated organizations, or those of the publisher, the editors and the reviewers. Any product that may be evaluated in this article, or claim that may be made by its manufacturer, is not guaranteed or endorsed by the publisher.

## Abbreviations

CM-DASH: Chinese Modified Dietary Approaches to Stop Hypertension
GEE: generalized estimation equation
SBP: systolic blood pressure
DBP: diastolic blood pressure
